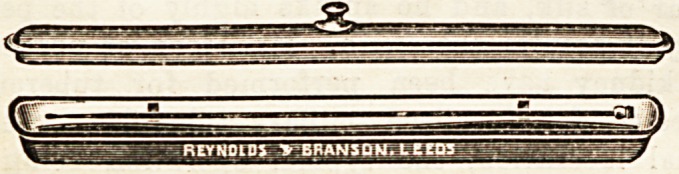# New Appliances and Things Medical

**Published:** 1898-07-23

**Authors:** 


### NEW APPLIANCES AND THINGS MEDICAL.

[We shall be glad to receive, at our Office, 28 & 29, Southampton Street, Strand, London, W.C., from the manufacturers, specimens of all new preparations and appliances which may be brought out from time to time.]

### A NEW CATHETER DISH.

Devised by Mr. H. de Paiva B. Veale, late house surgeon, General Infirmary, Leeds.

(Messrs. Reynolds and Branson, Limited, Leeds.)

The accompanying illustration shows a dish designed for the reception of flexible catheters in daily use. It consists of an elongated narrow vessel, furnished with an overlapping lid, and it is sufficiently long to admit a catheter of ordinary length without flexion being necessary. The catheter rests on two transverse ridges attached to the bottom of the dish, so that the antiseptic solution with which the dish is filled surrounds the instrument. A flexible catheter once having been used has to be bent to be placed in most of the receptacles as used at present, and this frequently produces "kinking," or injury at the eye. In this dish the catheter can be kept perfectly straight, and a minimum amount of solution is required to keep it submerged. The vessel being long and flat it may be kept by a patient's bedside without fear of upsetting it, and the shallow depth, combined with the absence of angles renders the dish very easily cleansed and sterilised. A label may be attached to the handle, or the patient's name may be written directly in ink or pencil on the lid. The dishes will serve for all forms of flexible catheters or bougies, and may also be used for drainage tubes. They are manufactured of iron, enamel ware, glass, or porcelain.

## Figures and Tables

**Figure f1:**